# Food price policies improve diet quality while increasing socioeconomic inequalities in nutrition

**DOI:** 10.1186/1479-5868-11-66

**Published:** 2014-05-20

**Authors:** Nicole Darmon, Anne Lacroix, Laurent Muller, Bernard Ruffieux

**Affiliations:** 1 INRA, NORT UMR1260, F-13385 Marseille, France; 2 INSERM, NORT UMR1062, F-13385 Marseille, France; 3 INRA, GAEL UMR 1215, F-38000 Grenoble, France; 4 Univ. Grenoble Alpes, GAEL UMR 1215, F-38000 Grenoble, France

**Keywords:** Experimental economics, Nutrition policy, Nutritional quality, Food choices, Poverty

## Abstract

**Background:**

Prices are an important determinant of food choices. Consequently, food price policies (subsidies and/or taxes) are proposed to improve the nutritional quality of diets. The aim of the present study was to explore the impact of food price policies on the expenditures and nutritional quality of the food baskets chosen by low- and medium-income households.

**Methods:**

Experimental economics was used to examine two price manipulations: *i)* a fruit and vegetable price subsidy named “fruit and vegetables condition”; *ii)* a healthy-product subsidy coupled with an unhealthy-product tax named “nutrient profile condition”. The nutrient profiling system called SAIN,LIM was used. This system classifies each individual food according to its overall nutritional quality which then allows for a food item to be taxed or subsidized. Women from low- (n = 95) and medium-incomes (n = 33) selected a daily food basket, first, at current prices and then at manipulated prices. The redistributive effects of experimental conditions were assessed by comparing the extent of savings induced by subsidies and of costs generated by the tax on the two income groups. Energy density (kcal/100 g), free sugars (% energy) and the mean adequacy ratio (MAR) were used as nutritional quality indicators.

**Results:**

At baseline (before price manipulations), low-income women selected less expensive and less healthy baskets than medium-income ones. After price manipulations expenditures for both income group decreased significantly, whereas, the nutritional quality improved (energy density decreased, the MAR increased). Additionally, the redistributive effects were less favourable for low-income women and their nutritional quality improvements from baseline were significantly lower.

**Conclusion:**

Low-income women derived fewer financial and nutritional benefits from implemented food subsidies and taxes than medium-income women. This outcome suggests that food price policies may improve diet quality while increasing socio-economic inequalities in nutrition.

## Background

Poor diets and low physical activity levels are among the main cause of obesity and excess risks of non-communicable diseases, including cardiovascular disease, hypertension, type 2 diabetes, stroke and certain cancers [1,2]. Price is a determining factor in food choices [3] and unhealthy eating is more prevalent among people with a low socio-economic status [4]. In particular, low-income individuals consume less fruit and vegetables and more refined cereals than high income ones. Such socioeconomic differences in food choices have been explained by the economic costs of a healthier way of eating [5]. In France, as many as 12.5% of adults are suffering from food insufficiency defined as "household getting enough, but not always the kinds of food they want to eat; or sometimes or often not getting enough to eat, for financial reasons" [6]. On average, their energy intake does not differ from food sufficient adults. The difference is in a lower intake of fruit and vegetables and a higher intake of high fat/high sugar foods leading to diets with poor nutritional quality (higher energy density, lower nutrient adequacy). Such unhealthy food choices could be due, at least in part, to food budget constraints [7], because high fat-high sugar foods are considerably cheaper sources of calories than fruit and vegetables and other healthy foods [8].

Changing relative food prices has therefore been proposed to improve the quality of diets [9-11]. Prices of foods high in calories, fat, or sugar may be increased in order to discourage their consumption and prices of healthy foods, such as fruit and vegetables, may be decreased to encourage consuming them. Such price manipulations could be implemented in the frame of public policies using specific taxes and subsidies. A vast literature has tried to assess the efficiency of such policies, nevertheless many methodological considerations and some weaknesses have been underlined. An extensive review [12] has concluded that studies estimating price effects on substitutions from unhealthy to healthy food, and price responsiveness among low-income populations were crucially needed. Simulation modeling studies have suggested that taxes based on a single nutrient or a single food tend to generate undesired effects on the demand for other nutrients or foods, due to substitutions between taxed and non-taxed foods, and due to heterogeneous consumer responses depending on their income level [13-15]. In addition, it has been estimated that only moderate health gains can be achieved with policies aimed at increasing fruit and vegetable consumption, while a risk of increasing disparities in health has been identified [16].

In a recent review of food price interventions tested either in real settings (cafeteria, restaurants) or in the laboratory, Epstein et al. concluded that food price policies were able to modify the purchases of targeted foods but that the net impact on nutritional quality was difficult to assess because of unknown possible substitution effects [17]. Experiments conducted in real settings have shown that price changes were able to positively influence the purchases of isolated food products [18], though they did not address possible substitution or income effects. Only one [19] of the experiments reviewed by Epstein reported real market conditions. However, in this study, the effects of value size pricing were evaluated exclusively on fast food meal choices and not on a basket of foods, so that substitution effects could not be estimated. In all the other experiments reviewed, the participants reported the hypothetical purchase of products, often with a fixed budget constraint.

Hypothetical decision-making means that participants do not actually purchase any products; they are asked to declare what they would have purchased in the real world. However, declarative statements without any financial incentives can be tainted by social desirability bias [20]. Hence new kinds of experiments are needed to reveal true consumers preferences, and to better determine substitution and income effects when assessing the impact of a given policy on the overall nutritional quality of purchases.

Experimental economics places an individual under controlled conditions, as close as possible to the real world [21-23]. In this kind of experiment, participants make choices with real products and real money *i.e.* they actually select and purchase products in the lab. This incentive mechanism limits the social desirability bias [24]. The standard procedure used in experimental economics generally observes one choice among a set of possible products [25]. In the present study, an innovative procedure was used: a set of choices for a full basket of food was observed. This protocol allowed us to compare the impact of food price manipulations on the nutritional quality, cost and food content of individual daily food baskets selected by low-income and medium-income women. Nutrient profiling, which classifies individual foods according to their overall nutritional quality [26], was used to decide which foods should be taxed or subsidized.

## Methods

### Participants

A convenience sample of 160 women was recruited, mainly selected according to their household income, more precisely, according to their disposable income per consumption unit. Disposable income was gross household income after deduction of direct taxes and payment of social security contributions. Household income was adjusted for household size by assuming an equivalent scale of 1 for the first adult, 0.5 for other individuals over 14 and 0.3 for children under 14. Two income groups were targeted: *i)* a low-income group which was the core of the study and included women who earned less than 60% of the French median disposable income per consumption unit, *i.e.* below the poverty line; *ii)* a control group, referred to below as ‘medium-income group’, whose disposable incomes per consumption unit ranged between the fourth and the seventh deciles of the French population. The women with the lowest income were approached through specialized institutions – health clinics, charity grocery stores, and other charities – and the others via a recruitment agency. Along with the income criterion, three other eligibility criteria for the participants were *i)* age 20-54; *ii)* grocery shopper for self or household; *iii)* use of a French food repertoire.

The experiment took place between 20 May and 26 July 2008, in six suburbs of Grenoble and Lyon, in South-Eastern France. Each woman received €25 to compensate her for the participation.

The sample consisted of 128 women, after eliminating participants who had incomplete responses and those whose income level did not correspond to the criteria that we had set:

– 95 belonged to the low-income group. Their disposable incomes per consumption unit were an average of €6,864 (9,288 USD; 5,792 GBP) per annum with a minimum of €3,156 (4,270 USD; 2,663 GBP) and a maximum of €9,672 (13,088 USD; 8,162 GBP).

– 33 women belonged to the medium-income group. Their disposable incomes per consumption unit were an average of €18,000 (24,360 USD; 15,190 GBP) per annum, with a minimum of €12,636 (17,100 USD; 10,664 GBP) and a maximum of €20,004 (27,070 USD; 16,881 GBP).

The two sub-samples sizes were sufficient to detect a difference of 0.5 of a standard deviation between zero and the mean difference of the pairs, with 95% confidence interval and power of 80%.

## Materials

The food composition database was originally developed for the Su-Vi-Max project. It gives the nutritional content (39 components) of 923 food items [27]. A total of 180 food items commonly purchased by French adults were used for this experiment (excluding alcoholic beverages).

For each selected food item, actual retail prices (referred to below as ‘observed prices’) were taken from the largest French cyber food market (Ooshop) in May 2008. Participants were informed of this source before the experiment.

The software package [28], originally developed for food consumption surveys, was adapted to the present purpose. Using it, a participant could compose an individual food basket by ‘picking’ products on a screen from a tree-structured database. Once a product was chosen, the portion was selected according to pictures.

### Nutrient profiling of foods and price manipulations

The decision to tax or to subsidize foods was based on their nutritional composition using the previously validated French SAIN,LIM nutrient profiling system [29,30]. This system is based on two independent scores calculated for each food: the SAIN (Score of Nutritional Adequacy of Individual foods) and the LIM (score of the nutrients which should be LIMited in a healthy diet).

The positive SAIN sub-score is the mean nutrient adequacy percentage calculated per 100 kcal for 5 basic nutrients (proteins, fibre, vitamin C, iron and calcium) along with a variable number of optional nutrients. The negative LIM sub-score is the mean percentage of maximal recommended values for 3 nutrients whose intake should be limited: saturated fatty acids, added sugar and sodium, calculated per 100 grams. Using the SAIN and LIM thresholds, foods were allocated to 3 classes: high SAIN and low LIM (healthy products); low SAIN and low LIM, or high SAIN and high LIM (neutral products); low SAIN and high LIM (unhealthy products). Among the class of healthy products, fruit and vegetables were distinguished from “other healthy products”. Fruit and vegetables included fresh fruit and vegetables, fruit juices, soup and canned or frozen vegetables. Potatoes, nuts and processed fruit containing added sugars (canned fruit in syrup, compote, fruit drinks) were excluded from this category.

According to the SAIN,LIM system, lean meat, eggs, milk, low fat dairy products, most fish and shellfish fell under the "other healthy products" category while refined cereals, potatoes, drinking water, fruit drinks, and canned fruit fell under the class of neutral products. Virtually all sweets and desserts, animal fats, sweetened beverages, a high proportion of salted snacks and mixed dishes, and most deli meats fell under the class of unhealthy products. Eventually, 43 foods were classified as “fruit and vegetables”, 24 foods as “other healthy products”, and 51 and 62 foods as neutral and unhealthy foods, respectively.

Two different price manipulations were tested: the ‘fruit and vegetable condition’ (FV condition) consisting of a 30% decrease in the observed price of fruit and vegetables, and the ‘nutrient profile condition’ (NP condition) consisting of a 30% decrease in the observed price of fruit and vegetables and of other healthy products and of a 30% increase in the observed prices of unhealthy products.

### Experiment implementation and data collection

Prior to the experiment, participants answered a questionnaire with socio-demographic data, including occupation, income and household size. Then the experiment consisted in four steps described in Table 1. In the first step, called “learning”, each participant was asked to describe the previous day’s daily food intake by selecting a ‘day-before food basket’ using the food-purchasing software. One of the purposes of this 24 h recall was to familiarize participants with the software. Subsequently, the participants were asked to select a ‘day-after food basket’ by selecting all the food that they intended to consume over the next 24 hours. Three alternative ‘day-after food baskets’ were successively selected. In the first one, called “baseline’, food items were posted at observed prices (*i.e.* no price manipulation). For the other two food baskets, the observed prices were modified in order to test first the FV condition and then the NP condition. The rationale of the alteration of prices was not explained to the participants. They could, however, identify the products concerned as changed prices appeared on the screen next to the old one, which was crossed out.

**Table 1:** The steps of the experiment

	***Learning***	***Baseline***	***Fruit and vegetable condition***	***Nutrient profile condition***
	***(24 h recall)***			
**Task**	Day-before daily food basket per person	Day-after daily food basket per person	Day-after daily food basket per person	Day-after daily food basket per person
**Prices**	No posted price	’Observed prices’	-30% fruit and vegetables	-30% healthy foods (including fruit and vegetables) + 30% unhealthy foods
**Data collection**		Dietary indices	Dietary indices	Dietary indices
		Expenditures	Expenditures, redistributive effects	Expenditures, redistributive effects

The participants were informed that their food choices would generate real sales. A subset of the 180 real products, hidden from them during the experiment, was placed in the room adjacent to the experimentation room. For all of a subject’s choices corresponding both to the available subset of real products and to a food basket randomly selected among the three composed of by the participant, the actual portions chosen were bought at the end of the session. Each woman paid for the purchases and went back home with the products.

At the end of the experiment, participants had to fill out another questionnaire on their eating habits, height, weight, and any chronic health disorders. Stated height and weight were used to calculate BMI and to identify overweight individuals (BMI ≥ 25). Considering health disorders the participants were asked three questions: “Do you suffer from high blood pressure? Do you suffer from cholesterol? Do you suffer from diabetes?”

### Data analyses

The impact of price manipulations was measured for low-income and medium-income groups based on the difference between the food basket selected at baseline and the one selected under each experimental condition. The impact was analysed in terms of: *i)* total quantities of food (beverages included) for each class (fruit and vegetables, other healthy foods, neutral products and unhealthy products); *ii)* quantities of beverages only; *iii)* expenditures *i.e.* all the selected food valued at posted prices and *iv)* dietary quality indices.

The energy density (in kcal/100 g) of diets was used as an indicator of bad nutritional quality because diets with a low energy density have been shown to have good overall nutritional quality [31,32]. The weight (in g) and energy content (in kcal) of food were calculated for each basket by adding up the edible weight and the energy content of selected foods. As proposed by Ledikwe et al. [33], only items typically consumed as foods, including soups, were included in the calculation. Beverages were excluded. A recent report from the World Research Cancer Fund recommends that the energy density of a diet (calculated without considering beverages) remain below 125 kcal/100 g [34].

The Mean Adequacy Ratio (MAR) was used as an indicator of good nutritional quality, as it has repeatedly been shown to be positively associated with other indices of dietary quality [35-37] and with health indicators [38,39]. MAR is a truncated index of the percentage of recommended intakes for several key nutrients [40]. By construction, the highest theoretically achievable value for the MAR is 100% adequacy/day. A total of 16 positive nutrients were included in the MAR, including fibre, proteins, vitamins, minerals, and essential fatty acids [41]. All selected foods (including milk, juice, and soft drinks) were used to calculate the MAR for each basket.

The free sugar content was expressed as an energy percentage. Free sugars were computed according to the WHO definition [1]: added sugars plus sugars naturally present in honey, syrup and fruit juices. The WHO recommends that free sugars provide less than 10% of total energy intake [1].

A general linear model (GLM) analysis was used to compare the means of each variable after adjustment for the total energy content of the selected daily basket. All analyses were stratified by income group and values were compared. An α level of 0.05 was used to determine statistical significance. Statistical analyses were performed using SAS software version 9.1 (SAS Institute, Cary, NC).

The redistributive effects of experimental conditions were assessed, through the comparison of savings induced by subsidies and of costs imposed by the tax among the two income groups. Savings were calculated for subsidized healthy products as differences between hypothetical expenditures at ’observed prices’ and expenditures under the tested conditions; costs were calculated for taxed unhealthy foods as differences between hypothetical expenditures at observed prices and actual expenditures.

In order to assess the impact of each experimental condition on nutritional inequalities, the differences induced were compared between the two income groups. This test, called the ‘Δ test’, was based on a comparison of the mean variations of each nutritional quality indicator between the baseline and each experimental condition, according to the income groups.

## Results

### Demographic characteristics and baseline food purchases

Table 2 shows the demographic characteristics of the two groups of participants. It highlights significant differences in levels of education, household size and employment, corresponding to differences of income levels between groups. It should be noted that income level (more precisely the disposable income per consumption unit) is dependent on employment, education level and household size; so the income group appears to be the most structuring variable of these socio-demographic differences. A higher prevalence of overweight problems and chronic health disorders was observed for low-income women, however neither difference was statistically significant. The likelihood of practicing sport was significantly higher among medium-income women. The energy content of the food baskets presented no significant differences between the income groups. The food baskets selected by low-income women, compared with those selected by the medium-income group, tended to contain a lower amount of fruit and vegetables (p = 0.0776) and a higher quantity of unhealthy products (p = 0.0631); these differences were not due to beverage quantity differences. They also contained a lower amount of “other healthy products” (p = 0.0008), including a lower amount of healthy beverages (p = 0.0127). The low-income women spent less than medium-income women on fruit and vegetables (p = 0.0373) and on other healthy products (p = 0.0065). They also tended to spend more on unhealthy foods (p = 0.093).

**Table 2:** Demographic and health characteristic of participants; energy and food contents of the individual daily food baskets (mean ± standard deviation or %)

	**Low-income group**	**Medium-income group**	**p-value**^**v**^
	**N = 95**	**N = 33**	
**Demographic characteristics**			
Age, y	35.3 ± 7.0	34.8 ± 6.9	0.3509
Education^i^, y	4.6 ± 2.1	6.5 ± 2.2	0.0001
Employed, %	17	70	<.0001
Income, € per month and per consumption unit	572.5 ± 140.4	1500.7 ± 444.5	<.0001
Household size, number of persons	3.8 ± 1.6	2.8 ± 1.4	0.0011
**Health characteristics**			
Body Mass Index^i i^, kg/m^2^	25.3 ± 5.8	23.9 ± 4.3	0.1921
Overweight, %	44	33	0.2744
At least one chronic health disorder^i i i^, %	20	12	0.3098
Never sport, %	58	24	0.0149
**Total energy content**, kcal			
Learning (24 h recall)	1487.48 ± 584.6	1458.28 ± 679.7	0.4957
Baseline	1555.69 ± 949.7	1456.16 ± 643.5	0.4546
Fruit and vegetable condition	1471.33 ± 575.4	1409.98 ± 698.2	0.2926
Nutrient profile condition	1400.19 ± 570.5	1364.06 ± 550.5	0.3085
**Food content at baseline**^**iv**^, in grams			
Fruit and vegetables (beverages included)	410.3 ± 380.8	514.7 ± 362.6	0.0776
*Beverages alone*	*56.3 ± 133.9*	*38.2 ± 78.3*	*0.3381*
Other healthy products (beverages included)	518.7 ± 290.1	703.6 ± 325.2	0.0008
*Beverages alone*	*342.1 ± 249*	*462.5 ± 318.9*	*0.0127*
Neutral products (beverages included)	727.5 ± 454.1	903.0 ± 664.8	0.1285
*Beverages alone*	*495.5 ± 389.6*	*630.7 ± 665*	*0.3729*
Unhealthy products (beverages included)	322.9 ± 395.8	195.9 ± 235.5	0.0631
*Beverages alone*	*66.3 ± 157.2*	*39.3 ± 123*	*0.3242*
**Expenditures**^**iv**^, €			
Fruit and vegetables	1.16 ± 0.91	1.57 ± 1.20	0.0373
Other healthy products	1.23 ± 1.10	1.70 ± 1.22	0.0065
Neutral products	1.27 ± 2.13	1.42 ± 1.24	0.1539
Unhealthy products	1.56 ± 2.45	0.94 ± 1.26	0.0930

^i^the number of additional years of compulsory schooling in France (*i.e.* beyond 16 years).

^i i^calculated from height and weight stated by participants.

^i i i^as stated by participants.

^iv^except for fruit and vegetables, foods are classified as other healthy, neutral and unhealthy products according a nutrient profiling described in the Method section. The values reported there are crude values *i.e.* not energy-adjusted.

^v^Wilcoxon rank sum test or Pearson χ^2^ test against null hypothesis of no differences between income groups.

### Impact of price manipulations on food purchases

**Figure 1:**
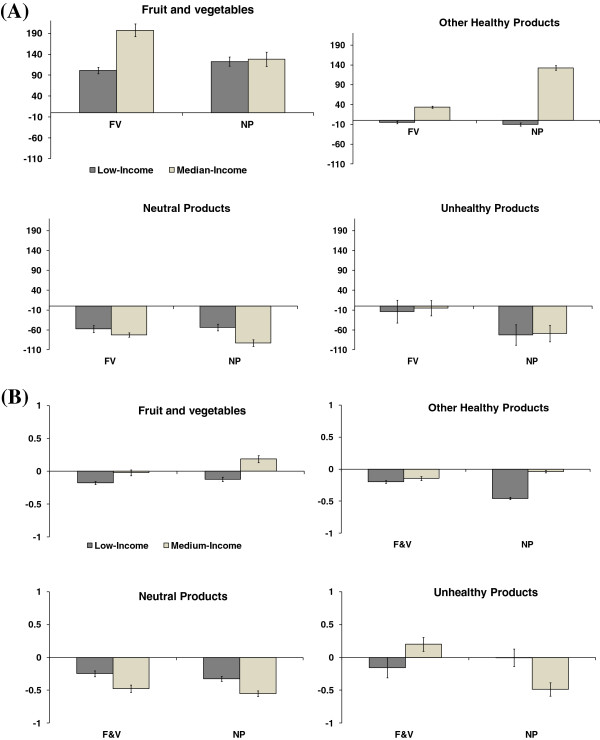
**Average variations of food at baseline and under price manipulations for low- and medium-income women. (A)** Quantities (grams per day). FV: fruit and vegetable condition, NP: nutrient profile condition. Note: Values are the mean of weight variations (grams per day) between the quantity selected at baseline and the quantity selected under the price manipulation, adjusted for the total energy content of the selected daily basket. Bars represent the standard error of mean calculated for α=0.05. **(B)** Expenditures (€ per day). FV: fruit and vegetable condition, NP: nutrient profile condition. Note: Values are the mean of expenditures variations (€ per day) between the food selected at baseline and the food selected under the price manipulation, adjusted for the total energy content of the selected daily basket. Bars represent the standard error of mean calculated for α=0.05.

Figure 1-panel A shows that the quantities of fruit and vegetables purchased were significantly increased by both price manipulations; this effect happened in both income groups (+25% for low-income group and +38% for middle-income group with FV condition; respectively +30% and +25% with NP condition). Figure 1-panel B shows that women in the low-income group reduced their fruit and vegetable expenditures under both the FV condition (-15%) and the NP condition (-10%). The results were different for the middle-income women: their fruit and vegetable expenditures were stable under the FV condition and increased under the NP condition (+12%). The quantities of other healthy products only increased for the medium-income group and the magnitude of this increase was greater with the NP condition (+19%) than with the FV condition (+5%). The amount spent for other healthy products decreased under each condition for low-income women, and decreased only under the FV condition for medium-income ones. Quantities and expenditures of neutral products significantly decreased in both conditions in both income groups.

The quantity of unhealthy products decreased only under the NP condition. Despite the same reduction between income-groups the quantities of unhealthy products purchased by the low-income group were still twice as much as those purchased by women in the medium income group (data not shown). Ultimately low-income women’s expenditures for unhealthy products remained the same, whereas these expenditures were significantly reduced in the medium-income group after the NP condition (- 52%).

Table 3 presents the values of expenditures, redistributive effects and diet quality indicators at baseline and under each price manipulation for the two income groups. At baseline, the food baskets selected by low-income women were significantly less costly and less healthy (higher energy density, lower MAR, more free sugars) than those selected by medium-income women.

**Table 3:** Individual daily food baskets and redistributive effects at baseline and under price manipulations

	**Low-income (LI) group**	**Medium-income (MI) group**	**Difference LI-MI**	
	**n = 95**	**n = 33**		**Δ test**^**2**^
**Expenditures,** € per day				
Baseline	5.23 ± 2.2	5.62 ± 1.5	-0.39 [0.0154]	
Fruit and vegetable condition	4.45 ± 1.6 (<.0001)	5.18 ± 1.9 (0.0123)	-0.73 [0.0369]	0.2643
Nutrient profile condition	4.34 ± 1.9 (<.0001)	4.73 ± 1.9 (<.0001)	-0.39 [0.1665]	0.2331
**Redistributive effects**^**1**^**,** € per day				
Fruit and vegetable condition	0.42 ± 0.3	0.66 ± 0.5	-0.24 [0.001]	n.a.
Nutrient profile condition	0.41 ± 0.8	1.36 ± 1.0	-0.95 [<.0001]	n.a.
**Energy Density,** kcal per 100 g				
Baseline	141.82 ± 32.5	122.61 ± 22.0	19.21 [0.0003]	
Fruit and vegetable condition	134.29 ± 22.2 (0.0147)	106.40 ± 27.0 (<.0001)	27.89 [<.0001]	0.0002
Nutrient profile condition	131.51 ± 17.94 (<.0001)	107.21 ± 17.31 (<.0001)	24.30 [<.0001]	0.0022
**Mean Adequacy Ratio,** % adequacy per day				
Baseline	55.23 ± 14.2	61.12 ± 9.7	-5.89 [0.0002]	
Fruit and vegetable condition	58.44 ± 11.0 (<.0001)	63.41. ± 13.4 (0.0457)	-4.97 [ 0.0409]	0.0577
Nutrient profile condition	56.54 ± 12.1 (0.0210)	63.35 ± 11.7 (0.0406)	-6.81 [0.0020]	0.4588
**Free sugars,** % energy				
Baseline	12.5 ± 2.3	11.09 ± 1.59	1.41 [0.0003]	
Fruit and vegetable condition	13.03 ± 2 (<.0001)	10.07 ± 2.43 (<.0001)	2.96 [<.0001]	<.0001
Nutrient profile condition	12.6 ± 1.9 (0.1256)	10.91 ± 1.88 (0.0989)	1.69 [<.0001]	0.0259

^1^crude values.

^2^test for difference between groups of average changes from baseline values.

Note: values are the mean ± standard deviation and, in brackets, p-value against null hypothesis of difference from baseline or between income groups (Wilcoxon test). Except for redistributive effects, values are energy-adjusted.

Both price manipulations significantly reduced expenditures for both income groups. The expenditure difference between the two income groups was still significant under the FV condition, but was no longer present under the NP condition.

At the end of each price manipulation, purchases of subsidized healthy foods by low-income women were smaller than those of medium-income ones; at the same time, low-income women still purchased two time more taxed unhealthy products than medium-income women. As low-income women purchased less subsidized foods and more taxed foods, they benefited less from subsidies and paid more taxes than medium-income women. Redistributive effects were therefore less favourable for low- income women than for wealthier ones.

Both price manipulations significantly improved the energy density and the MAR for both income groups but the energy density improvement was significantly greater for medium-income than for low-income women. Whatever the condition, the two indices remained less favourable for low-income women than for the medium-income women.

The NP condition had no significant impact on energy contributed by free sugars. The FV condition had limited effects, with opposite outcomes in the two income groups: an improvement (*i.e*. a reduction) for the medium-income group and degradation (*i.e*. an increase) for the low-income one. This degradation could be due to an increase of sugar content of beverages and especially of fruit juices. As this increase is not statistically significant, no conclusion can be drawn. Further examination of the food content in the baskets (results not shown) revealed that, among low-income women, the FV condition increased the quantities of all kinds of food products containing fruit and vegetables, even those that were not subsidized (fruit drinks, canned fruit in syrup containing free sugars). Further examination of nutrient content in the baskets (see Tables 4 and 5) almost always displayed improvements under both price manipulations: nutrients specific to fruit and vegetables, such as fiber, vitamin C, vitamin A, potassium and folates increased under both price manipulations and for both income groups; nutrients of concern, such as cholesterol, saturated fatty acids and sodium either decreased (as expected) or did not change; other micronutrients either slightly increased or did not change.

**Table 4:** Nutrients content (mean +/- standard deviation) of individual daily food baskets at baseline and under price manipulations for low-income group (n=95)

	**Baseline**			**Fruit and vegetable condition (FV)**			**Nutrient profile condition (NP)**			**FV/Baseline**	**NP/Baseline**
										**(1)**	**(1)**
Proteins (g/d)	66.90	+/-	31.5	64.82	+/-	19.5	59.91	+/-	20.7	0.4496	0.0016
Fat (g/d)	62.60	+/-	41.7	56.49	+/-	24.5	55.81	+/-	24.2	0.588	0.206
Cholesterol (mg/d)	307.51	+/-	151.8	268.93	+/-	84.3	301.62	+/-	118.4	0.0131	0.7485
PUFA* (g/d)	9.11	+/-	5.9	7.93	+/-	3.2	7.67	+/-	2.8	0.1866	0.0151
MUFA* (g/d)	23.47	+/-	16.2	20.94	+/-	8.9	20.26	+/-	8.8	0.6241	0.0763
SFA* (g/d)	25.56	+/-	16.8	23.56	+/-	10.7	23.73	+/-	10.5	0.7767	0.6401
Carbohydrates (g/d)	181.36	+/-	112.3	176.11	+/-	69.3	164.61	+/-	67.6	0.2339	0.2578
Added sugars (g/d)	46.48	+/-	44.3	41.64	+/-	21.0	38.37	+/-	20.3	0.5285	0.2694
Fibers (g/d)	14.83	+/-	5.8	15.57	+/-	4.8	15.38	+/-	5.3	0.0016	0.0376
Ca (mg/d)	674.40	+/-	254.7	712.58	+/-	168.9	677.76	+/-	167.4	<0001	0.1135
Fe (mg/d)	8.50	+/-	4.6	8.64	+/-	2.8	8.62	+/-	3.1	0.0149	0.0797
Mg (mg/d)	221.15	+/-	83.0	223.22	+/-	57.3	218.77	+/-	60.8	0.0377	0.4986
K (mg/d)	2198.43	+/-	709.2	2312.69	+/-	530.6	2227.03	+/-	608.2	<0001	0.1306
Na (mg/d)	2939.60	+/-	1138.4	2700.27	+/-	849.6	2945.52	+/-	764.5	0.0312	0.1563
Vitamin A (mg/d)	983.69	+/-	229.2	1151.17	+/-	331.1	1262.73	+/-	343.6	<0001	<0001
Thiamin (mg/d)	0.86	+/-	0.4	0.94	+/-	0.3	0.86	+/-	0.3	<0001	0.2594
Riboflavin (md/d)	1.22	+/-	0.5	1.26	+/-	0.3	1.23	+/-	0.4	0.0022	0.191
Niacin (mg/d)	12.30	+/-	5.4	12.21	+/-	3.6	11.44	+/-	4.1	0.1355	0.1099
Vitamin B6 (mg/d)	1.20	+/-	0.4	1.30	+/-	0.3	1.30	+/-	0.4	<0001	<0001
Folates (mg/d)	225.50	+/-	76.4	259.52	+/-	50.0	264.95	+/-	63.1	<0001	<0001
Vitamin B12 (μg/d)	3.93	+/-	1.1	3.56	+/-	0.8	3.85	+/-	0.8	<0001	0.7691
Vitamin C (mg/d)	82.02	+/-	17.4	114.32	+/-	30.8	112.56	+/-	35.8	<0001	<0001
Vitamin D (μg/d)	1.36	+/-	0.8	1.35	+/-	0.5	1.31	+/-	0.7	0.1853	0.9139
Vitamin E (mg/d)	7.67	+/-	2.9	7.04	+/-	1.9	7.16	+/-	1.8	0.0448	0.0982

values are energy adjusted.

(1) p-value against null hypothesis difference from baseline (Wicoxon test).

*PUFA: polyunsaturated fatty acids, MUFA: monounsaturated fatty acids, SFA: saturated fatty acids.

**Table 5:** Nutrients content (mean +/- standard deviation) of individual daily food baskets at baseline and under price manipulations for medium-income group (n=33)

	**Baseline**			**Fruit and vegetable condition (FV)**			**Nutrient profile condition (NP)**			**FV/Baseline**	**NP/Baseline**
										**(1)**	**(1)**
Proteins (g/d)	73.81	+/-	21.3	72.26	+/-	23.7	72.52	+/-	20.0	0.4999	0.3944
Fat (g/d)	54.88	+/-	28.3	52.06	+/-	29.7	46.74	+/-	23.3	0.4247	0.0005
Cholesterol (mg/d)	340.32	+/-	102.9	290.31	+/-	102.4	284.85	+/-	114.2	<0001	<0001
PUFA* (g/d)	7.85	+/-	4.0	7.47	+/-	3.9	6.61	+/-	2.7	0.6557	0.0005
MUFA* (g/d)	20.70	+/-	11.0	18.58	+/-	10.8	17.10	+/-	8.5	0.0509	0.0002
SFA* (g/d)	22.26	+/-	11.4	22.19	+/-	12.9	19.45	+/-	10.1	0.8682	0.001
Carbohydrates (g/d)	166.99	+/-	76.1	163.23	+/-	84.1	163.40	+/-	65.2	0.7598	0.6557
Added sugars (g/d)	37.23	+/-	30.0	32.81	+/-	25.5	35.98	+/-	19.6	0.2785	0.7866
Fibers (g/d)	15.44	+/-	4.0	17.94	+/-	5.9	17.67	+/-	5.1	0.032	<0001
Ca (mg/d)	727.83	+/-	172.6	750.68	+/-	204.9	745.03	+/-	161.5	0.1532	0.1696
Fe (mg/d)	9.18	+/-	3.1	9.10	+/-	3.4	8.91	+/-	3.0	<0001	0.1997
Mg (mg/d)	236.54	+/-	56.2	271.13	+/-	69.5	269.89	+/-	58.7	0.1151	<0001
K (mg/d)	2448.22	+/-	480.5	2699.63	+/-	643.9	2661.15	+/-	586.8	<0001	<0001
Na (mg/d)	2929.25	+/-	771.3	2895.61	+/-	1031.0	2706.49	+/-	737.6	0.0607	0.0002
Vitamin A (mg/d)	772.99	+/-	155.3	1216.07	+/-	401.8	1115.96	+/-	331.5	<0001	<0001
Thiamin (mg/d)	1.01	+/-	0.3	1.07	+/-	0.4	0.94	+/-	0.3	0.2478	0.0031
Riboflavin (md/d)	1.47	+/-	0.4	1.46	+/-	0.4	1.45	+/-	0.4	0.882	0.4999
Niacin (mg/d)	14.40	+/-	3.7	14.47	+/-	4.4	14.35	+/-	3.9	0.7465	0.6812
Vitamin B6 (mg/d)	1.40	+/-	0.3	1.58	+/-	0.4	1.55	+/-	0.4	<0001	<0001
Folates (mg/d)	246.22	+/-	51.8	296.66	+/-	60.7	283.38	+/-	60.9	<0001	<0001
Vitamin B12 (μg/d)	4.62	+/-	0.7	4.28	+/-	1.0	5.19	+/-	0.8	0.0005	<0001
Vitamin C (mg/d)	82.29	+/-	11.8	124.15	+/-	37.4	119.49	+/-	34.6	<0001	<0001
Vitamin D (μg/d)	1.96	+/-	0.5	1.02	+/-	0.7	1.28	+/-	0.6	<0001	<0001
Vitamin E (mg/d)	7.20	+/-	2.0	7.57	+/-	2.3	7.23	+/-	1.8	0.0249	0.6941

values are energy adjusted.

(1) p-value against null hypothesis difference from baseline (Wicoxon test).

*PUFA: polyunsaturated fatty acids, MUFA: monounsaturated fatty acids, SFA: saturated fatty acids.

## Discussion

The present results confirm that the food choices of low-income consumers are less healthy than those of medium-income consumers [42-46]. In addition, the results show that the two food price manipulations tested, *i.e.* subsidizing fruit and vegetables (FV condition) or subsidizing healthy products while taxing unhealthy ones (NP condition), were able to improve some aspects of the nutritional quality of food choices in both income groups. They especially reduced the energy density of the individual daily food baskets selected by low-income and medium-income women. Doing so they tend to reduce the total energy content of the baskets as the decrease of energy density is positively correlated with a reduction in overall calorie intake [47-49]. However, the dietary quality differences observed at baseline between the two income groups were still present or even increased under both conditions, which suggests that it would be difficult to fight social inequalities in nutrition with the food price policies investigated in this research. Additionally, the implemented price manipulations were regressive: the low-income group derived fewer financial and nutritional benefits from the conditions than the medium- income group.

Low-income women purchased less fruit, vegetables and other healthy products, and more unhealthy products, than medium-income women. These food choice differences observed at baseline between the two groups of women were associated with significant differences in the quality of their diet: energy density and free sugars were significantly higher, and the MAR was significantly lower for low-income women. In accordance with the literature [4], the present study did not show income-related differences for energy content but noted significant differences for micronutrients (as assessed by the MAR) and for energy density.

An important finding of the present study was that none of the tested price manipulations were able to reduce the income-related inequalities in nutrition; on the contrary, they may even have increased them. Favourable expected impacts were recorded for both income groups, although the extent of the improvement was always greater for the medium-income group. Other unwanted impacts of the experimental conditions need to be mentioned. First, both conditions induced a decrease in the purchasing of neutral products (mainly staple foods such as pasta, rice, bread …), indicating that the increase of subsidized products is not necessarily done at the expense of unhealthy products. The quantities of neutral products decreased even at the end of the price manipulations, neutral products remained among the cheapest products. This reduction suggests that the demand for staple foods decreased in favour of more expensive foods, since economic conditions allowed consumers to do so. Second, the substitutions for costlier products were limited as both conditions induced reductions of total food expenditures. This may indicate that, in real life, women would take advantage of the opportunity to reduce their food budget in order to increase expenditures for other items such as clothing, leisure or any non-food items. Third, low-income women were not as affected to price modifications as medium-income women. This result would indicate that the poor have less flexible food consumption patterns, in accordance with the food monotony associated with poverty [50].

Some limitations of the method used in the present study must be noted. Even though the participants were not informed of the nutritional objectives of the experiment, presenting the products with the new price next to the old crossed-out price might have induced price comparison and therefore might have influenced behaviour leading to the avoidance of unhealthy products in favour of healthy ones. Yet the incentive mechanisms of experimental economics, *i.e.* the obligation to buy the products chosen at the end of the session, limit this social desirability bias. In the present experiment, the amount spent by each participant (around €5) was close to the average expenditure for food at home in France (€5.8 per person and per day at the time of the experiment [51]), suggesting that the incentive was sufficient enough to reveal the participants’ true preferences. The second limitation stems from the nature of the samples, which are convenience samples selected on four criteria and thus not representative samples. However, beyond the major eligibility criterion (income level) these samples presented the expected socio-economic differences in terms of education, occupation, and sport practice. A third limitation stems from the experimental context itself. This enables us to reach our objective of controlling the environment and reducing noise in the lab, but in doing so we may also highlight the change of context. As a consequence, part of the observed effects on altered behaviours may either not exist in a noisy real life context, or not likely to be sustainable in the long term.

In spite of such methodological limitations the results showing an increase in fruit and vegetable purchases when they are subsidized, are convergent with those of other experimental studies [17]. Many simulation studies using price elasticity data derived from econometric calculations showed that taxing unhealthy foods has negligible effects on the nutritional quality of the whole diet [52-55]; meanwhile, food tax combined with appropriate subsidies could be more efficient [56,57]. Our results confirm the effectiveness of a policy which consists of simultaneously subsidizing healthy products and taxing unhealthy ones. Combining food taxes with subsidies was also considered as a good way to alleviate potential regressivity “by enabling consumers to switch to more healthy products without incurring additional costs” [58]. However, we have shown here that such a price manipulation may increase income disparities in financial and nutritional benefits. From this point of view, our results converge with Nordstrom and Thunstrom’s modelling showing that tax reforms aimed at improving dietary quality seem to have a positive health effect across all income groups except the lowest income one [59].

Our results do not converge with the conclusion of the two studies which stated that the beneficial nutritional effects of food tax reforms are more pronounced for low-income earners [15,57]. Two reasons may explain the discrepancy between our experimental study and these econometric studies. First, in our study low-income earners were among the poorest deciles of incomes of the French population and were therefore probably poorer than people from the lowest social class (among five) in the Danish study [15] and people from the lowest income quintile in the British study [57]. Second, these studies considered an aggregate representative consumer and did not take into account all differences in individual preferences. Nnoham et al. [57] assumed that price elasticities are the same for all income groups. Both studies did not consider that some individuals do not consume one or more of the foods. A recent study [60] bypassed these limitations assuming a more complex model of demand. In the present study, food choices were recorded for each individual and for each price condition. Therefore the heterogeneity of preferences among consumers and individual food substitutions in response to price changes were directly observed; whereas, in econometric studies, they were dependent on assumptions.

Overall, our study shows that price policies that attempt to alter individual food behaviours would not be effective in reducing social inequalities in nutrition. In this respect our results are consistent with the findings of Frohlich and Potvin [61] showing that improving the health of the overall population may increase health disparities between social groups: those who were formerly at a lower exposure to risk derive more benefits than those who were formerly at a greater exposure to risk. Given the widening gap in socio-economic inequalities in health in Europe [62], including in France [63], more research is needed on the possible differential impacts of a food tax reform on individuals, depending on their socioeconomic positions and incomes.

## Conclusion

The present study suggests that food price policies *i.e.* fruit and vegetable subsidies and food tax combined with appropriate subsidies may improve some aspects of diet quality. However it must be kept in mind that laboratory results are indicative: laboratory experiment conditions give the price manipulations the best chance to succeed owing to the extent of the price variations and the saliency of the behavioral variations. Despite these ideal conditions, the 30% price manipulations induced limited dietary changes. In addition, since wide-ranging taxes and subsidies were selected in this study, it is unlikely that actual policies would use such high rates, which could be judged politically unacceptable.

More importantly, our results showed that the low-income group derived fewer financial and nutritional benefits than the medium-income group from both price manipulations. Therefore, the present study suggests that price policies are regressive and may increase social inequalities in food consumption and dietary quality. It is a good argument for targeting policies at low income groups.

## Competing interests

The authors declare that they have no competing interests.

## Authors' contributions

BR initiated the study and participated in and supervised all its aspects. ND, AL, LM and BR participated in the design of the study. AL, LM and BR conducted the data collection. ND and AL wrote the article. All of the authors participated in data analysis, and read and approved the final manuscript.

## Authors' information

Nicole Darmon is a nutritionist (PhD) from the “Nutrition, Obesity and Risk of Thrombosis” research unit at INRA (National Institute for Agricultural Research), Aix-Marseille University.

Anne Lacroix and Laurent Muller are economists (PhD), working for INRA, and Bernard Ruffieux is Professor of Economics at the school of Industrial Management at the Grenoble Institute of Technology. These three authors are currently developing their research in the ‘Grenoble Applied Economics Laboratory’ (INRA and Grenoble Alpes University).
